# Progressive familial intrahepatic cholestasis type‐3 and multiple sclerosis: lessons from comorbidity

**DOI:** 10.1002/acn3.50883

**Published:** 2019-09-30

**Authors:** Roberto De Masi, Stefania Orlando, Antonella De Donno

**Affiliations:** ^1^ Laboratory of Neuroproteomics Multiple Sclerosis Centre “F. Ferrari” Hospital 73042 Casarano – Lecce Italy; ^2^ Complex Operative Unit of Neurology “F. Ferrari” Hospital 73042 Casarano – Lecce Italy; ^3^ Laboratory of Hygiene, Department of Biological and Environmental Sciences and Technologies University of the Salento Lecce Italy

## Abstract

The comorbidity between multiple sclerosis (MS) and progressive familial intrahepatic cholestasis type‐3 (PFIC3) has never been described yet. ABCB4 gene encodes the multidrug resistant protein 3 (MDR3) and its mutations induce PFIC3 as well as intrahepatic cholestasis of pregnancy (ICP) and drug‐induced liver injury (DILI). We describe the case of a 32‐year‐old female with MS and PFIC3 who was effectively treated with natalizumab and ursodeoxycholic acid (UCDA), in contrast to glatiramer acetate, dimethylfumarate, and IFNb1a associated with DILI. Our findings clarify the pharmacodynamics of MS therapies and suggest natalizumab plus UDCA as the effective treatment of PFIC3/MS phenotype, unlike the others that should be avoided.

## Introduction

The timely recognition of comorbidities in clinical practice is paramount to individualize effective treatment and to minimize adverse effects in treating multiple sclerosis (MS).^1^ In particular, the comorbidity between MS and progressive familial intrahepatic cholestasis type‐3 (PFIC3) has never been described to date.

PFIC represents a heterogeneous group of autosomal recessive cholestasis diseases of childhood. Mutations in the same genes responsible for PFIC lead to other nonprogressive cholestasis diseases in adults, including intrahepatic cholestasis of pregnancy (ICP) and drug‐induced liver injury (DILI).

The underlying gene that is defective in PFIC3 is ABCB4 (7q21) that encodes the multidrug resistance protein 3 (MDR3).^2^ MDR3 is a 12‐domain transmembrane plasmalemmal translocator, responsible for actively flipping phospholipids, particularly phosphatidylcholine, on the interface hepatocyte‐bile ducts. This molecule, belonging to the P‐glycoprotein (P‐gp) superfamily of translocases, is expressed at the blood–brain barrier and secretory/absorptive tissues such as the gastrointestinal tract, kidneys, and liver.

Furthermore, MDR3 can also bind a number of synthetic molecules, conferring resistance to chemotherapy, even though worldwide efforts to identify its selective inhibitors have not been successful.^3^

## Case Presentation

P. L., a 32‐year‐old woman, was admitted to our neurological department in May 2012, presenting with right face and arm paresis that had started about 6 days before. MRI brain demonstrated contrast enhancing and nonenhancing lesions in the periventricular and juxtacortical white matter sites, typical for MS.

The isoelectric focusing of the cerebrospinal fluid (CSF) showed seven oligoclonal bands not expressed in the serum. CSF showed normal protein concentration and the link index was 1.2, without pleocytosis, suggesting blood–brain barrier breakdown process.

Blood test for MS mimics was negative and the patient was diagnosed with relapsing remitting MS according to the 2011 McDonald criteria. She underwent treatment with 5 days of intravenous high‐dose methylprednisolone.

At the hospital discharge, we recommended as first‐line MS therapy three times weekly subcutaneous administration of glatiramer acetate, as well as follow‐up every 3 months at the local MS center. The expanded disability status scale (EDSS), improved by one point compared to baseline, was 2.0. Routine blood tests were normal.

During the first few weeks of treatment, the patient experienced a moderate increase in liver transaminases, to about four to five times the normal values (n.v.). Three months after starting therapy, the patient had an intense allergic reaction, with breathing difficulties and a diffuse rash. Glatiramer acetate was discontinued in favor of three times weekly subcutaneous administrations of high‐dose IFNb1a. However, elevated transaminases persisted, with a moderate increase in level, even after switching to low‐dose weekly administration of IFNb1a 3 months later.

At this point, the patient was lost to the follow‐up for about 2 years, after which she reappeared in our department with two children in apparent good health.

Medical records provided evidence of elevated transaminases of five to six times the n.v. and ICP during both pregnancies. By then, however, the woman had only a mild increase of transaminases, about twice the n.v., with stable neurological conditions and no jaundice or pruritus. Six months after starting dimethylfumarate (orally 240 mg bid), increase of transaminases was moderate, but a brain MRI revealed a significant increase in lesion load (LL) with respect to the pretreatment baseline. The EDSS remained stable. Autoimmune, viral, alcohol, and storage liver diseases were excluded. We suspected a diagnosis of PFIC due to the history of DILI and ICP, and the PFIC type‐3 was confirmed with a liver biopsy and specific genetic tests.

The genetic profile exhibited a double heterozygosis for the ABCB4 gene with the following mutations: p.Arg652Gly (c.1954A > G) and p.Ile237= (c.711A > T); moreover, an intronic variant of TJP2 that is p.Lys16Glu (c.46A > G).

**Figure 1:**
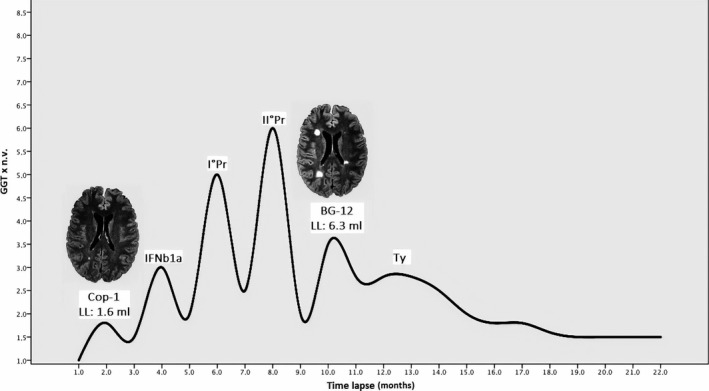
The graphic representation of the hepatic cytolytic indexes expressed as multiples of gamma‐glutamyl transferase (GGT) normal value (n.v.) during the first pregnancy (IPr), the second pregnancy (IIPr), and the drug administration: glatiramer acetate, interferon (IFNb1a), dimethylfumarate, and natalizumab (Ty). Note the magnetic resonance imaging of the brain with its lesion load (LL) expressed in milliliters (mL) at the start therapy and the last therapy before the natalizumab

**Figure 2:**
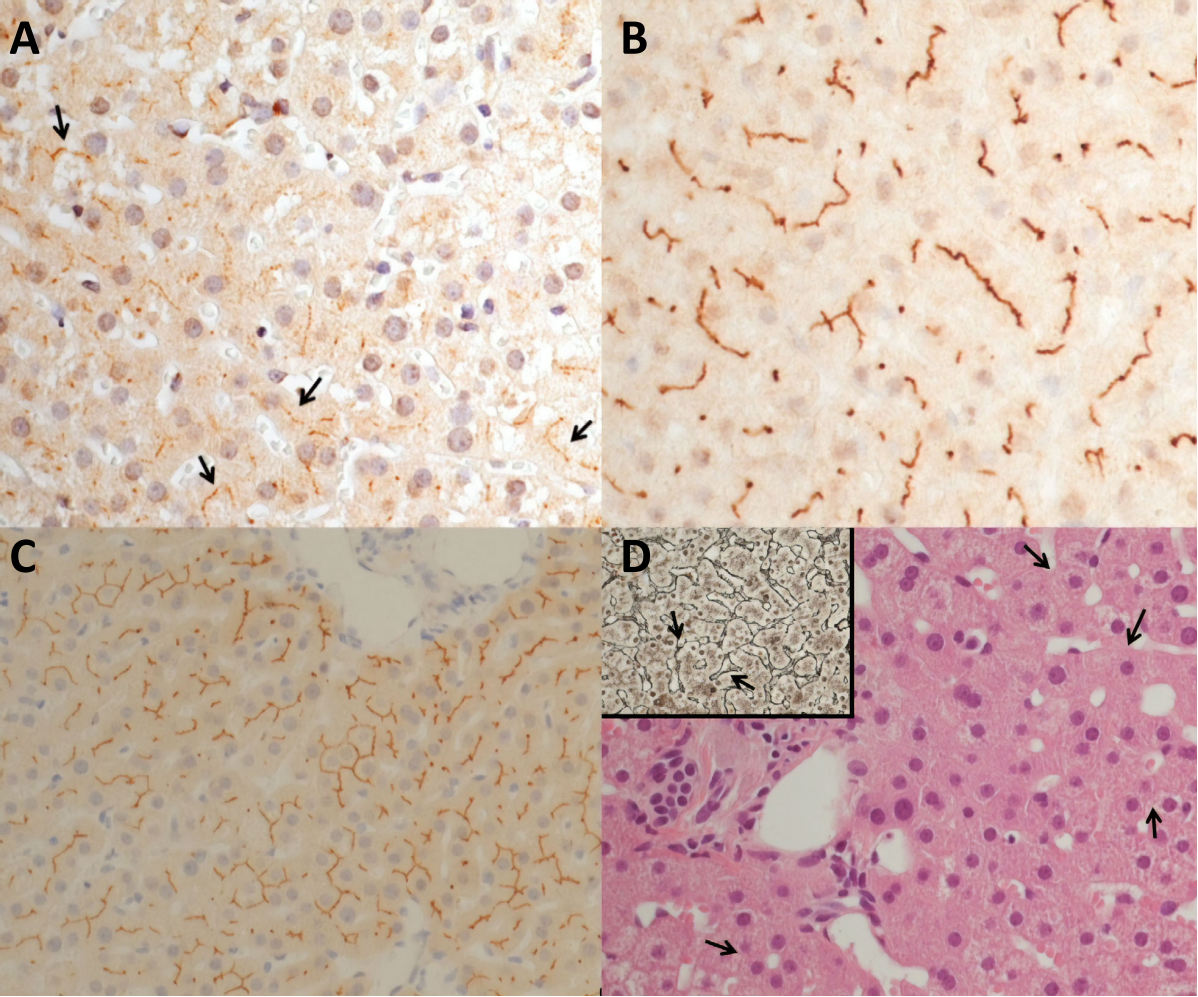
Immunohistochemical and histological staining of the liver biopsy. (A) focal loss of the multi‐drug resistant protein 3 (MDR3) at the hepatocytic ductal pole and its residual immunoreaction (black arrows); (B) MDR3 representation at the hepatocytic ductal pole in healthy control; (C) positive immunostaining of the bile salt export pump (BSEP); (D) the “hepatocytic rosette” in reticulus staining (black arrows in the little view) and hematoxylin‐eosin staining (black arrows in the large view)

**Figure 3:**
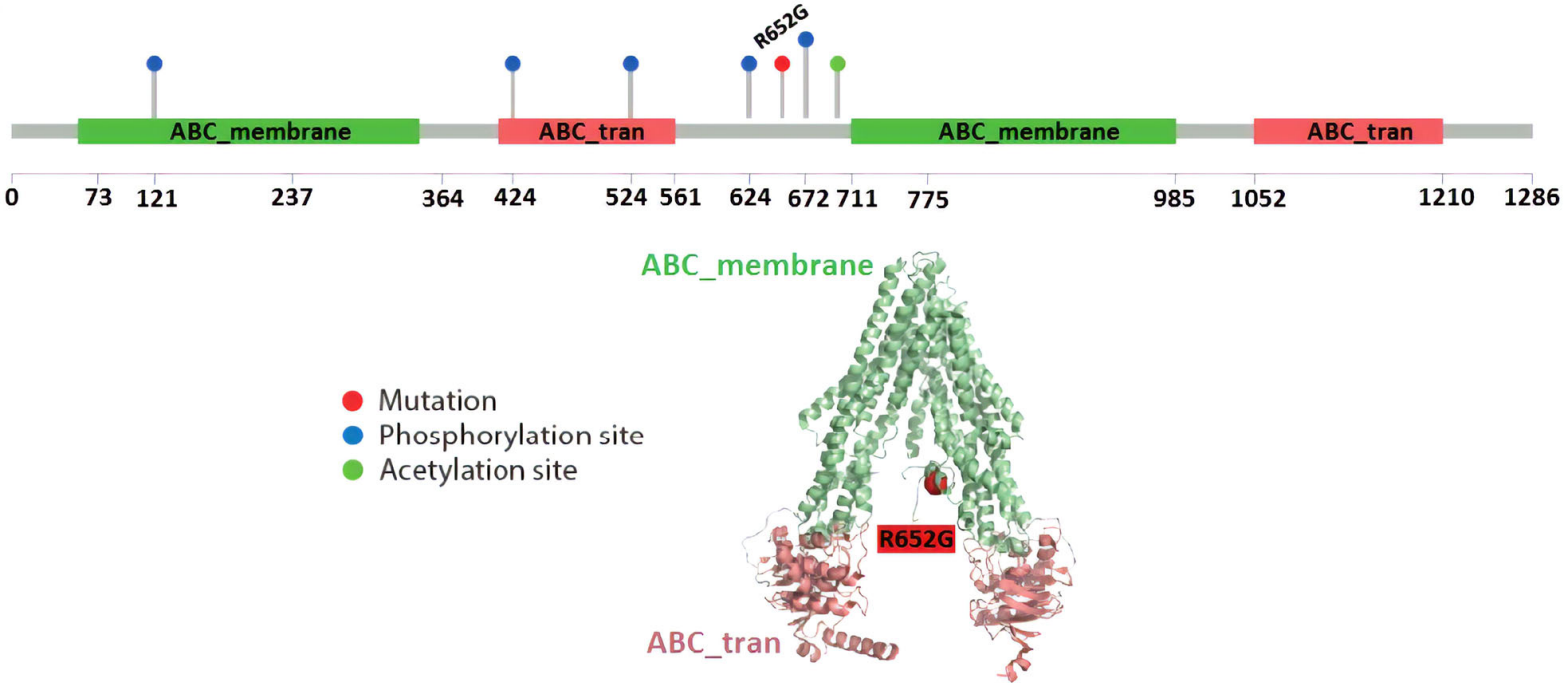
Protein sequence annotation of FIC‐selected nonsynonymous ABCB4 mutation displayed as red lollipop (R652G). Blue and green lollipops indicate phosphorylation and acetylation sites, respectively. Nonsynonymous mutation annotation on available 3D structure as assessed through Mechismo is displayed following the same color scheme regarding the ABCB4 gene

Figure 1 shows the LL and gamma‐glutamyl transferase (GGT) levels during the pregnancies and therapies. Note the increase of LL during the whole first‐line treatment period. Figure 2 shows the loss of MDR3, compared to the healthy control; positive immunostaining of the bile salt export pump (BSEP) and the “hepatocytic rosette”. In Figure 3, protein sequence annotation of FIC‐selected nonsynonymous mutation and annotation on available 3D structure are displayed (only for ABCB4, as TJP2 mutation map to unstructured region).

Finally, the patient was treated with oral ursodeoxycholic acid (UCDA) (10 mg/kg/die), plus natalizumab therapy. She continues to receive monthly infusions with unchanged MRI LL. Transaminases are now stable at 1.5–2 times the n.v., with no pruritus or jaundice.

## Discussion and Conclusions

We described a case of a MS patient with DILI and ICP history, who has ABCB4 involvement causing PFIC3, resulting in the PFIC3/MS clinical phenotype. Given its genomic basis, this comorbidity represents an “*experimentum naturae*”, because it explains the kinetics and dynamics of drugs tested in the mutant patient having MDR3 loss. Normal BSEP function excluded also the non‐PFIC conditions, as resulting by immunostaining. The drugs tested are disease‐modifying therapies (DMTs) that are considered first‐line (dimethylfumarate, glatiramer acetate, and IFNb1a) and second‐line therapies (natalizumab). DILI has been frequently described in literature, but its exact mechanisms are still not understood.^4^ For DMTs in MS, as with other drugs, liver toxicity is attributed to the direct effect of the therapeutic molecule, as well as its idiosyncratic or autoimmune effects, the frequency of which depends on the specific drug.

The cause of hepatic injury from DMTs is not known, but it may be reversible following drug discontinuation. In the case of interferon, it may be dose‐related. The postulated mechanism is an autoimmune reaction for IFNb1a and glatiramer acetate, and an idiosyncratic reaction for dimethylfumarate. For natalizumab, the mechanism of liver injury is probably also immunologically mediated, resulting from a dose‐dependent leukocytes compartmentalization into the bloodstream.^5^

Unlike these reversible conditions, the hepatotoxicity described here was not resolved completely at drug discontinuation, but was persistent nonprogressive.

The persistent DILI with reduced expression of MDR3 protein can be sustained by the lack of translocation of phosphatidylcholine across the canalicular membrane into bile, causing proliferation of bile ducts and altered cytoarchitecture resulting in “hepatocytic rosette”. On the other hand, the accentuation of hepatotoxicity during administration of IFNb1a, glatiramer acetate, and dimethylfumarate is caused by hepatocyte back accumulation of the unbound drug to translocator.

This study suggests that the therapeutic activity of natalizumab is not linked to MDR3 and is not associated with serum aminotransferase elevation, unlike IFNb1a, glatiramer acetate, and dimethylfumarate that all link MDR3, contributing to hepatotoxicity in PFIC3/MS phenotype. For these reasons, natalizumab plus UDCA should be considered in the future as first‐line therapy in patients with this comorbidity, while the other DMTs described here should be avoided.

## Author Contributions

Roberto De Masi was a major contributor in conception and design, acquisition of data, analysis, and interpretation of data of this manuscript. Stefania Orlando has been involved in drafting the manuscript and revising it critically for important intellectual content. Antonella De Donno has given final approval of the version to be published. All authors read and approved the final manuscript.

## Conflict of Interest

All authors declare no conflict of interest.
